# New Observations of the Effects of the Cytoplasm of *Aegilops kotschyi* Boiss. in Bread Wheat *Triticum aestivum* L.

**DOI:** 10.3390/genes15070855

**Published:** 2024-06-28

**Authors:** Chaolan Fan, Joanna Melonek, Adam J. Lukaszewski

**Affiliations:** 1 Triticeae Research Institute, Chengdu Campus, Sichuan Agricultural University, Chengdu 611130, China; chaolanfan@genetics.ac.cn; 2 Division of Plant Sciences, Research School of Biology, The Australian National University, Canberra 2601, Australia; joanna.melonek@uwa.edu.au; 3 Department of Botany and Plant Sciences, University of California, Riverside, CA 92521, USA

**Keywords:** cytoplasmic male sterility, fertility restoration, parthenogenesis, haploidy, double fertilization

## Abstract

The cytoplasm of *Aegilops kotschyi* is known for the induction of male sterility and haploidy in wheat. Both systems originally appeared rather simple, but manipulation of the standard chromosome constitution of the nuclear genome revealed additional interactions. This study shows that while there is little or no allelic variation at the main fertility restorer locus *Rf^multi^* on chromosome arm 1BS, additional genes may also be involved in the nuclear–mitochondrial genome interactions, affecting not only male fertility but also the growth rate, from pollen competition for fertilization and early endosperm divisions all the way to seed size and plant maturity. Some of these effects appear to be of a sporophytic nature; others are gametophytic. Induction of parthenogenesis by a rye inducer in conjunction with the *Ae. kotschyi* cytoplasm is well known. However, here we show that the cytoplasmic-nuclear interactions affect all aspects of double fertilization: producing maternal haploids from unfertilized eggs, diploids from fertilized eggs or synergids, embryo-less kernels, and fertilized eggs without fertilization of the double nucleus in the embryo sack. It is unclear how frequent the inducers of parthenogenesis are, as variation, if any, is obscured by suppressors present in the wheat genome. Genetic dissection of a single wheat accession revealed five distinct loci affecting the rate of maternal haploid production: four acting as suppressors and one as an enhancer. Only when the suppressing haplotypes are confirmed may it be possible to the identify genetic variation of haploidy inducers, map their position(s), and determine their nature and the mode of action.

## 1. Introduction

The cytoplasm of *Ae. kotschyi* is one of several cytoplasms among the wild relatives of wheat (Triticum sp.) capable of inducing cytoplasmic male sterility (cms) in wheat, both hexaploid and tetraploid [1,2,3,4,5,6]. Male sterility manifests itself only in the absence of the chromosome arm 1BS, such as in the wheat-rye translocation 1RS.1BL. So far, only one chromosome 1B of wheat tested, that of *Triticum spelta var. duhamelianum*, was found to be incapable of restoring fertility; all other chromosomes 1B are capable of restoring fertility [3,4]. As this locus on chromosome arm 1BS is capable of restoring male fertility to several different cytoplasms, it was named *Rf^multi^* by Tsunewaki [7].

Cms based on the *Ae. kotschyi* cytoplasm would be of considerable value in wheat breeding, as under standard growing conditions and with the standard chromosome constitution of the nuclear genome, it does not appear to cause undesirable agronomic effects, and there are some indications that it may improve resistance to abiotic stresses [8]. The ubiquitous presence of a fertility restorer on 1BS simplifies fertility restoration in hybrids. However, fertility restoration by a single copy of *Rf^multi^* reaches only about 2/3 of a full seed set. Full fertility is restored only by two copies of the locus. Hence, additional selection/manipulation would be required for effective use of the system in hybrid breeding. One of the approaches was a transfer of *Rf^multi^* from its normal position on chromosome arm 1BS to rye chromosome arm 1RS and the translocation of that engineered 1RS arm to chromosome arms 1AL and 1DL [9]. Hybrids of male sterile wheats with wheats carrying such translocations are fully fertile as they carry two doses of *Rf^multi^*: one in its natural position on 1BS and the other on the 1RS.1AL or 1RS.1DL translocation. On the other hand, the ubiquitous *Rf^multi^* was removed from 1BS by chromosome engineering, creating three chromosomes: 1B_1:6_, 1B_25:6_, and 1B_35:6_ [9,10], to be used in the cms maintainer lines. Despite the absence of *Rf^multi^*, one of these engineered chromosomes started showing a low level of fertility restoration, suggesting that more than a single locus on 1BS may be involved.

With the chromosome constitution of the nuclear genome deviating from normal, the cytoplasm of *Ae. kotschyi* in wheat shows several additional effects pointing to a range of nuclear–mitochondrial interactions. One such effect is the rate of growth and grain size in the absence of the chromosome arm 1S in the B genome (either 1BS or 1RS). Such plants are always small and grow much more slowly than their sisters with 1BS present. They produce very small grains, as small as 1/10 of the normal test weight, and germinate poorly. Chromosome arm 1RS, in the wheat rye translocation 1RS.1BL, fully compensates the absence of 1BS. As *Rf^multi^* is absent from 1RS, some rye genes on 1RS must interact with the mitochondrial genome of *Ae. kotschyi*. The absence of other group 1S arms, such as 1AS or 1DS, has no discernible effect on the growth rate or seed size.

Even more interestingly, the cytoplasm of *Ae. kotschyi* is one of several cytoplasms among wheat relatives capable of affecting various aspects of double fertilization in wheat, resulting in the production of haploids (parthenogenesis), twin seedlings (mostly haplo-diplo), and embryo-less kernels [1,11]. This occurs when the proper inducer is present in the genome of an alloplasmic wheat line. The first such inducer discovered was a centric wheat–rye chromosome translocation 1RS.1BL in a wheat line ‘Salmon’, a hexaploid derivative of an octoploid triticale [12]. The haploid frequency induced by the Salmon source can exceed 90% [4].

Haploids are very interesting and useful organisms. Upon chromosome doubling, they produce homozygous doubled haploids. These are of considerable value in genetic research, especially for mapping quantitative trait loci (QTL) in biparental populations, and in practical breeding, as they eliminate the long process of genotype stabilization after the initial hybridization, hence the speeding up of the breeding process [13]. For these reasons, numerous approaches have been invented and improved to produce haploids on a large scale, mostly focused on the induction of microspores into the sporophytic pathway of development [14]. Genetic means of haploid production would be even more beneficial, as it would eliminate the need for tissue/cell culture. The combination of the *Ae. kotschyi* cytoplasm + 1RS inducer in wheat generates normally developed grain with normal germination rate but with some proportion of haploids or twin seedlings, of which one is usually haploid; a small proportion of seed is germless. For these three reasons: male sterility and fertility restoration, the effect on growth rate, and haploid production, this system attracted the attention of the authors, with the idea of identifying the loci responsible for the three phenomena, but specifically the 1RS locus responsible for the induction of parthenogenesis.

Genetic mapping to locate a locus of interest requires polymorphism (allelic variation). Apart from the single case of non-restoring 1BS arm of wheat described by Mukai and Tsunewaki [3], no polymorphism for the *Rf^multi^* appears to be present. The locus was located on the 1BS by Tsunewaki [7] using a set of 1RS-1BS recombinant lines, later re-located, re-mapped, and renamed by Chen [15], and sequenced [16]. The availability of the wheat IWGSC reference genome, pan-genome data sets, and the reference genome of rye *S. cereale* “Lo7” enabled studies of the genomic interval carrying *Rf^multi^* in wheat and the corresponding non-restoring region in rye [16,17,18]. These comparative studies pointed to several candidate genes in the region, including *TraesCS1B02G071642.1* [16,17] and TraesCS1B01G072300 [18]. Both candidate genes have a strong prediction for mitochondrial localization and belong to the P subclass of the pentatricopeptide repeat protein family, to which the majority of identified restorer genes in crops belong [19]. The candidate gene mapped by Chen [15] on 1BS in wheat and named Rfk1 appeared to be *TraesCS1B02G197400LC*. Later, the same authors proposed the same gene as a candidate for Rfd1 restorer gene for the *Ae. juvenalis* cytoplasm [20]. The *TraesCS1B02G197400LC* gene encodes a pectin esterase inhibitor located to the cell wall. How a cell wall-localized pectin inhibitor protein interacts with the cms-causing genes encoded in the mitochondrial genomes remains to be studied. 

To the best knowledge of the authors, no attempt was ever made to locate genetic loci on 1BS associated with the growth rate of alloplasmic wheat lines with the cytoplasm of *Ae. kotschyi*. As 1RS fully compensates for the absence of 1BS, recombinants 1RS-1BS are useless for this purpose and this aspect of the study remains unattended. This left the parthenogenesis-inducing factor(s) on 1RS as the primary focus. In search of allelic variants, a set of 1RS chromosome arms originating from various sources, all in a uniform genetic background of cv. ‘Pavon 76’, were crossed and backcrossed to an alloplasmic line of cv. Pavon with the cytoplasm of *Ae. kotschyi*. No haploids were ever detected, either in 1RS disomics in or heterozygotes with 1BS. At the same time, by fortuitous coincidence, high frequencies of haploids were detected in an alloplasmic line of triticale cv. (*kot*)Presto with the 1D(1B) substitution and in an alloplasmic line of a winter wheat cv. (*kot*)Joker. Cv. ‘Joker’ carries the wheat-rye translocation 1RS.1BL which does not appear to be related to the most common version of the translocation from cvs. ‘Aurora’ and ‘Kavkaz’ or that of Salmon (F.J. Zeller, personal communication). 

When chromosome arm 1RS from cv. Presto, already present in cv. Pavon, both as a substitution 1R(1B) or translocation 1RS.1BL, was tested in the cytoplasm of *Ae. kotschyi*, no haploids were detected. Suspecting an error during line development, chromosome 1R from Presto 1D(1B) and translocation 1RS.1BL from cv. Joker were transferred into (*kot*)Pavon by backcrosses, and again, no haploids were detected. This indicated that cv. Pavon carries a suppressor or suppressors of parthenogenesis somewhere in the genome, but not on chromosome arm 1BS. Both the 1R(1B) substitution and 1RS.1BL translocation remove the 1BS arm. Mukai [11] indicated that chromosome arm 1BS of wheat carries such a suppressor; it appeared to operate gametophytically (no 1B carrying eggs produced haploids) and did not completely suppress haploid production by a heterozygote 1BS + 1RS.1BL. Data by Mukai [11] also hint at a non-1BS suppressor or suppressors: cvs. Aurora and Kavkaz differed dramatically in haploid production, with the former exceeding 90% when homozygous. Both of these cultivars carry 1RS.1BL translocation, which, by all indications, originated from the same source [21] and appear to be identical [22], suggesting that the absence of haploids in (*kot*)Kavkaz was not caused by the absence of the inducer (on 1RS), but by the presence of some other factor suppressing their production. The presence of a parthenogenesis suppressor in cv. ‘Chinese Spring’ or an enhancer in Salmon was speculated on by Tsunewaki and Mukai [4].

Taken together, these observations raise questions about the nature of haploid induction by the 1RS arm in the *Ae. kotschyi* cytoplasm: Are inducers, such as those in Salmon, Joker, and Presto, indeed rare, or are non-1BS suppressors of haploid production in the wheat genome common? Zeller and Hsam [21] state but provide no direct evidence or reference that “most European 1B/1R wheat cultivars transferred into the cytoplasm of *Ae. kotschyi* are also able to produce haploids”. The 1B/1R cultivars in the cited segment reflect the old notation of either 1R(1B) substitution or 1RS.1BL translocation.

This study was undertaken to address the issues of fertility restoration to the *Ae. kotschyi* cytoplasm, including a search for allelic variants of *Rf^multi^*, to identify and locate in the genome of wheat a suppressor or suppressors of haploid production, and to establish a haplotype/haplotypes associated with the absence of suppressors, so that allelic variation of the haploidy inducers can be tested and their genetic location determined. 

## 2. Materials and Methods

### 2.1. Plant Materials

**Figure 1:**
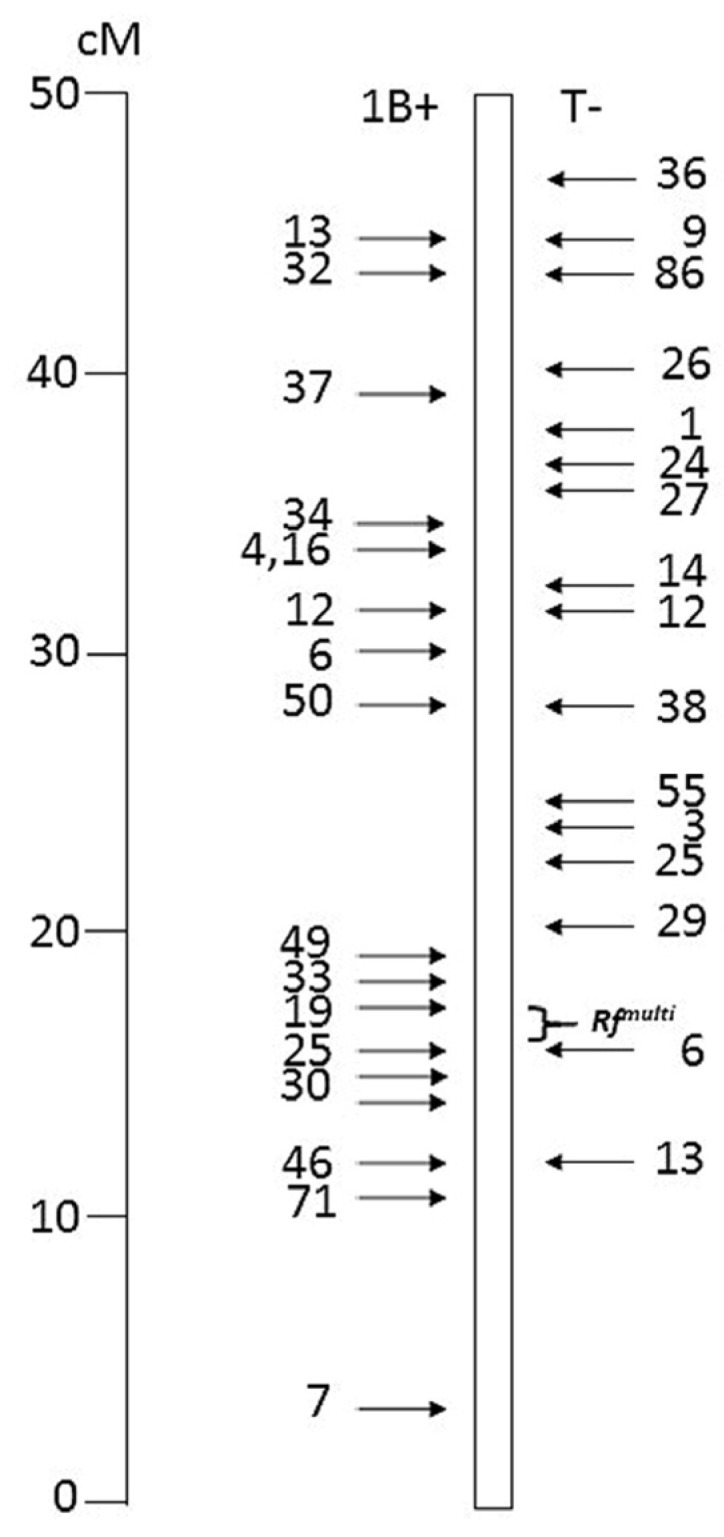
Distribution of the 33 translocation breakpoints in 1BS-1RS recombinants tested in this study, based on their genetic positions. On the left, configuration 1B+, with distal rye and proximal wheat segments; on the right, configuration T- with distal wheat and proximal rye segments. Telomere on top. The *Rf^multi^* interval is marked.

All chromosome constructs used in this study were developed previously, for several different purposes. The *ph1b*-induced recombinants of 1BS with 1RS used here (Figure 1) originate from the set described by Lukaszewski [23] and characterized by Fan et al. [24]. Cytogenetically engineered chromosomes 1B, with rye chromatin inserts removing *Rf^multi^*, and chromosomes 1RS, with wheat chromatin inserts carrying *Rf^multi^*, were described by Hohn and Lukaszewski [10] and Lukaszewski [9]. Centric translocations 5RS.5AL and 2BS.2RL are from AJL’s collection; the latter was described and characterized by Lukaszewski et al. [25] and Fan et al. [24]. All of these stocks are in the genetic background of wheat cv. Pavon 76, a Spring white wheat from the International Maize and Wheat Improvement Center (CIMMYT), El Batan, Mexico, originally provided to the author by Dr. R. Rajaram, from now on referred to as Pavon. The alloplasmic line of cv. Chinese Spring with the cytoplasm of *Ae. kotschyi* was provided by Prof. K. Tsunewaki, then at the Fukui Prefectual University, Fukui, Japan, while a wheat line K116A with the *Ae. kotschyi* cytoplasm was provided by Dr. P.S. Baenziger, then at the University of Nebraska, Lincoln NE USA. Both lines were crossed and repeatedly backcrossed to cv. Pavon, among others, which currently are in the BC_7_ and BC_11_ generations, respectively. The Pavon lines with *Ae. kotschyi* cytoplasm are male fertile; male sterility was obtained by cross-pollination with Pavon 1RS.1BL or Pavon Dt1BL lines. The standard 1RS arm in Pavon 1RS.1BL originates from cv. Kavkaz, via CIMMYT’s cv. ‘Genaro’. All engineered chromosomes have the chromatin of this specific 1RS arm. Additionally, chromosome 1R, in substitution for 1B or as translocation 1RS.1BL, was transferred into Pavon from winter triticale (*xTriticosecale* Wittmack) cv. Presto by backcrosses. Cv. Joker was among a set of European winter wheats provided for different experiments by Dr. Z. Banaszak, then at Danko Plant Breeding Ltd., Poland. The pedigree of cv. Joker is Chevalier/WW3640 (WW3640 = Carlos/Certo), with the 1RS.1BL translocation most likely originating from cv. Kronjuwel, a distant progenitor of cv. Certo, which has a triticale derivative in its pedigree (Dr. M. Koch, Deutsche Saatveredelung AG, pers. comm.). This translocation does not appear to be related to the 1RS.1BL translocation present in the Salmon wheat line or the Aurora/Kavkaz source (F.J. Zeller, personal comm.).

### 2.2. The Nomenclature

The nomenclature used in this article for the plasmon (cytoplasm) identification generally follows the system proposed by Tsunewaki [26] but is abbreviated to (*kot*)Pavon to indicate cv. Pavon with the plasmon of *Ae. kotschyi*. For clarity, euplasmic lines are denoted as, for example, (*aes*)Pavon. The origin of chromosome translocations or chromosome arms in translocations is denoted by a subscript, as in Lukaszewski [27], except that with the number of chromosomes from various sources growing, two or three letters are now required to identify each accession. For example, notation Pavon 1RS.1BL_jok_ indicates translocation 1RS.1BL from cv. Joker transferred to cv. Pavon, Pavon 1RS_ct_.1BL indicates 1RS arm from triticale Presto (original breeding line designation CT771/81 was retained) translocated to the 1BL arm of Pavon. All stocks had at least seven backcrosses completed, unless noted otherwise.

### 2.3. Growing Conditions

All materials were grown in a greenhouse at the University of California, Riverside campus, with two generations per year: one planted in late September and harvested in January, and the second planted in late February or March and harvested in June/July. The greenhouse has an evaporative cooling system; typical growing conditions include 16-h days/8-h nights provided by supplemental lights, with temperatures at 23–26 °C during the day and 15–18 °C at night for much of the season. All grain for analyses originated from bagged heads. Pollinations were performed by hand. All karyotyping was performed by C-banding, following the protocols in Lukaszewski and Xu [28].

### 2.4. Endosperm Development Test

For tests of the early endosperm divisions, heads were emasculated and bagged, and hand pollinated 3–4 days later. The time of each pollination was noted, and the heads were harvested and fixed in a mixture of 3 parts absolute ethanol–1 part glacial acetic acid 24, 48, and 72 h after pollination. For analyses, individual caryopses were hydrolyzed in 1N HCl at 60 °C for 10 min, stained with the Feulgen stain and dissected as in Bennet et al. [29].

### 2.5. Genetic Mapping

To produce the mapping population of potential suppressors of parthenogenesis in Pavon, a line of Pavon with the introgression of 1RS.1BL_jok_ after BC_5_ was crossed to Joker. The resulting F_1_ hybrid was backcrossed as male to (*kot*)Joker and the progeny were screened cytologically to select diploid homozygotes 1RS.1BL_jok_. Leaf samples were collected at the seedling stage from each diploid (6x), dried for several days above the silica gel and their DNA extracted using the protocol recommended by Diversity Arrays Technology, Canberra, Australia (https://www.diversityarrays.com/services/laboratory-services/dna-extraction/, accessed on 12 April 2024). All members of the mapping population were male sterile, as expected, and were cross-pollinated using pollen from various hexaploid wheat stocks producing pollen at that time. With several exceptions the seed set was high, approaching 100%, and of good quality. With the mistaken assumption of 1:1 segregation for high and no haploid production, in most cases only one head was cross-pollinated. Samples of seed from cross-pollination of the mapping population were germinated and screened for haploid frequency, by chromosome counts on standard root-tip squashes stained in acetocarmine. 

DNA extracted from the 131 diploid individuals in the mapping population was submitted to Diversity Arrays Technology Ltd. for genotyping using DArTseq at a level of 1.5 M reads. Markers polymorphic between Joker and Pavon were retained for the genetic map construction. Markers with more than 20% of missing scores were excluded. Markers were sorted into groups and ordered by the Kosambi distances by the MAP function of QTL IciMapping v4.1 (http://www.isbreeding.net/, accessed on 12 May 2019). The physical locations of markers in the Chinese Spring IWGSC reference genome version 2.1 [30] were used as anchoring information. Other parameters were set as default settings. To improve map quality, multiple rounds of ordering were executed followed by manual marker filtering. In each linkage group, subsequent markers with genetic distances beyond 50 cm were discarded and excluded from the next round of ordering. 

Genomic regions associated with haploid production were identified by the BIP function of QTL IciMapping v4.1 within the inclusive composite interval mapping (ICIM) analysis. The haploid frequencies of the mapping population members were used as the phenotype data. The logarithm of the odds (LOD) threshold was set at 2.5. Genomic regions delimited by markers were analyzed for the presence of candidate genes using the annotations available from the IWGSC 2.1 sequence [30] and the annotation repository hosted on the URGI website (https://wheat-urgi.versailles.inra.fr/Seq-Repository/Annotations, accessed on 12 April 2024).

## 3. Results

### 3.1. Fertility and Chromosome Transmission Rates

A single copy of the *Rf^multi^* locus in the (*kot*)Pavon line, such as in a heterozygote 1B + 1RS.1BL restores male fertility to ca. 2/3 of normal seed set (multiyear average of 2.0 vs. 3.1 seed per spikelet for euplasmic Pavon), hence these lines produce seed upon self-pollination. Surprisingly, no male sterile plants were ever recovered among such progenies when ca. 25% of such plants were expected. Karyotyping indicated that these progenies were composed of ca. 50:50 proportions of 1B disomics and 1B + 1RS.1BL heterozygotes. Male sterile progenies could only be obtained by backcrossing heterozygotes as female to Pavon 1RS.1BL or Dt1BL.

As described previously [9,10] the *Rf^mult^*^i^ locus on chromosome 1B was eliminated by inserts from rye, of three different lengths. While chromosomes 1B_25:6_ and 1B_35:6_ consistently produce male sterile plants in the *Ae. kotschyi* cytoplasm, chromosome 1B_1:6_ in some seasons shows some anther dehiscence and sets a few seeds per spike. To test if there are loci on the 1BS arm of Pavon other than Rf^multi^ which could be responsible for such a low level of fertility restoration, a set of 33 1RS-1BS recombinants with breakpoints distributed more or less evenly along the entire length of the genetic map of the arms.(Figure 1), were crossed to two (*kot*)Pavon lines. Recombinants with the *Rf^multi^* present were crossed to (*kot*)Pavon 1RS.1BL; those with *Rf^mult^*^i^ absent were crossed to (*kot*)Pavon. This way, both classes of the F_1_ hybrids had one copy of *Rf^mul^*^ti^, were male fertile and set seed upon self-pollination. Progenies were screened cytologically to select homozygotes for the recombinant chromosomes. Among 227 progeny from hybrids with 1B + non-*Rf^mult^*^i^ recombinants only one such homozygote was identified, for 1B+1. This is one of two primary recombinants used to create chromosome 1B_1:6_. There were 122 disomics 1B and 104 heterozygotes 1B + recombinant, indicating a strong selection against rye chromatin.

To test if the selection was against rye chromatin in general or against a specific region of 1RS, two wheat-rye centric translocations were tested, 2BS.2RL and 5RS.5AL, separately or in combination with 1RS.1BL. All were introduced into the *Ae. kotschyi* cytoplasm, by crosses to (*kot*)Pavon, *(kot*)Pavon 1RS.1BL, as well as (*aes*)Pavon and (*aes*)Pavon 1RS.1BL to serve as controls, and backcrossed as male and female to (*aes*)Pavon and (*kot*)Pavon. Samples of the resulting progenies were karyotyped. Full seed set in backcrosses using heterozygotes as female indicated the absence of gametic selection on the female side hence, only two samples each in two combinations were tested (Table 1). The female transmission rate of all three wheat-rye translocations was close to 50% and the cytoplasm of the egg played no role (Table 1). Not so on the pollen side: there was no transmission of the 1RS.1BL translocation via pollen with the *T. aestivum* cytoplasm; transmission of translocations 2BS.2RL and 5RS.5AL was normal, close to 50%. In pollen with the *T. aestivum* cytoplasm, all three translocations were transmitted normally, somewhat below 50%. Clearly, pollen with the *Ae. kotschyi* cytoplasm and the 1RS.1BL translocation suffered a serious competitive disadvantage.

To test if selection against 1RS in pollen with the *Ae. kotschyi* cytoplasm was general, or directed against a specific region of the arm, 16 recombinants 1RS-1BS with the *Rf^multi^* locus present (Figure 1) were crossed to (*kot*)Pavon 1RS.1BL. With one copy of the *Rf^multi^* present these were male fertile and their progenies from self-pollination were screened cytologically to identify homozygotes for the recombinants. Screened samples were small; only to recover two recombinant homozygotes. Among 66 progeny, there were 26 homozygotes for the recombinant chromosomes, 39 heterozygotes, and one homozygote 1RS.1BL, a serious deviation from the theoretical 1:2:1. Clearly, recombinants 1RS-1BS with *Rf^multi^* present were readily transmitted via pollen; complete 1RS arm was not. These transmission patterns indicated that pollen with the *Ae. kotschyi* cytoplasm and the *Rf^multi^* locus absent suffers a serious handicap in competition with similar pollen carrying 1BS with *Rf^multi^*. Pollen acetocarmine stainability test indicated a very low proportion (ca. 3–4% in several tests) of unstained clearly empty pollen (assumed to be non-viable) suggesting that pollen grains with both the *Rf^mul^*^ti^ present and absent are produced normally and both types are viable.

To further test the role of the *Rf^multi^* in transmission of chromosomes via pollen carrying *Ae. kotschyi* cytoplasm, chromosomes 1B_25:6_ and 1B_35:6_ [9] were combined with the standard translocation 1RS.1BL and with engineered chromosomes 1RS_33:34_.1BL and 1RS_49:34_.1BL [9]. Engineered 1BS arms have their *Rf^multi^* removed (1B*rf*); engineered 1RS have the *Rf^multi^* inserted (1RS*Rf*). In (*aes*)Pavon, the male transmission rate of such chromosomes 1B and 1R was normal, 1:1, regardless of the position of *Rf^multi^* (Table 2). In the cytoplasm of *Ae. kotschyi*, insertion of *Rf^multi^* into 1RS substantially improved its transmission rate against 1B, especially against 1B with *Rf^mult^*^i^ removed. However, in no case it offered it the same competitive advantage as a normal chromosome 1B had against normal 1R (Table 1). The same pattern was observed among progenies from self-pollination of heterozygotes. In (*aes*)Pavon, segregation of *1RSRf^multi^* vs. 1BS *Rf^multi^* was 25:59:30 (1RS”: heterozygote: 1BS”), not deviating from 1:2:1 but with a slight bias in favor of 1BS reflecting a somewhat reduced compensation of 1RS. The same combination of chromosomes in the cytoplasm of *Ae. kotschyi*, segregated 20:64:63, highly skewed against 1RS. To produce this segregation ratio, the male transmission of 1RS *Rf^multi^* must have been in the 17–19% range, almost three times higher than male transmission rate of normal 1RS vs. normal 1BS. When the same engineered 1RS arms carrying *Rf^multi^* were present in translocations 1RS.1AL or 1RS.1DL their segregation was normal, 1:2:1 (17:34:20) but again with some bias against 1RS. However, lines of Pavon with these two translocations were disomic for standard chromosome 1B with *Rf^multi^* present.

In five wheats other than cv. Pavon, grouped as “Assorted” in Table 2, the situation was more interesting: while there was selection against 1RS in competition with 1B (*rf* vs. *Rf*), it was not nearly as extreme as in Pavon: 50:28 in favor of 1B for the entire group. However, among these five wheats tested, cv. Renan stood out with very little selection against pollen with 1RS: 20:16 in favor of 1B. This suggests that chromosome arms 1BS from various wheats differ in their allelic content at loci interacting with the cytoplasm, and can either successfully outcompete 1RS in pollen transmission, as it is the case of 1BS from Pavon, or behave as neutral.

### 3.2. Growth Rate

**Figure 2:**
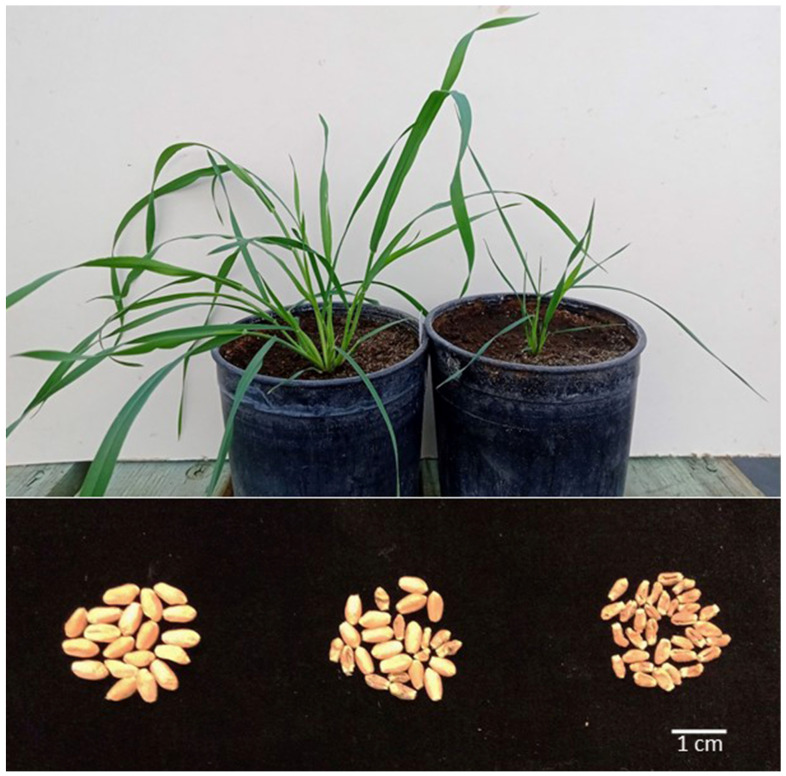
The effects of the 1S chromosome arm in the B genome of wheat with the cytoplasm of *Ae. kotschyi* on plant growth rate and seed size. Top: two sister plants (*kot*) Joker after BC_9_ ca. four weeks after the end of vernalization; on the left, normal growth; on the right, stunted growth, typical of missing the 1RS arm. Bottom: seed produced by plants (*kot*)Pavon (left), (*kot*)Pavon 1RS.1BL + 1BL (center) and (*kot*)Pavon Dt 1BL (right).

In the absence of wheat chromosome arm 1BS, or rye chromosome arm 1RS in the 1RS.1BL translocation (in essence, ditelocentric 1BL), the growth rate of the plants with the *Ae. kotschyi* cytoplasm is severely reduced (Figure 2). Heading and maturity are delayed, by up to four weeks under the UCR greenhouse conditions, and plants are approximately 1/3 the size of their counterparts with the *T. aestivum* cytoplasm. Such plants produce very small and shriveled seed (Figure 2) with a very low germination rate. Plants with a single copy of 1BS, such as monosomic 1B, 1B + 1BL, 1RS.1BL + 1BL or double monosomic 1R + 1B, produce two classes of seed: normal and small (Figure 2). Full size seeds germinate normally and as a general rule, carry either 1BS or 1RS, in whatever configuration either one was present in the maternal plant. The proportions of normal: small seed reflect the female transmission rate of the 1BS or 1RS arms, and so it is 50:50 for progenies of the combinations 1B + 1BL and 1RS.1BL + 1BL, ca. 25% for progenies of monosomics 1B, 1R or 1RS.1BL. This indicates that the control of seed size is gametophytic.

**Figure 3:**
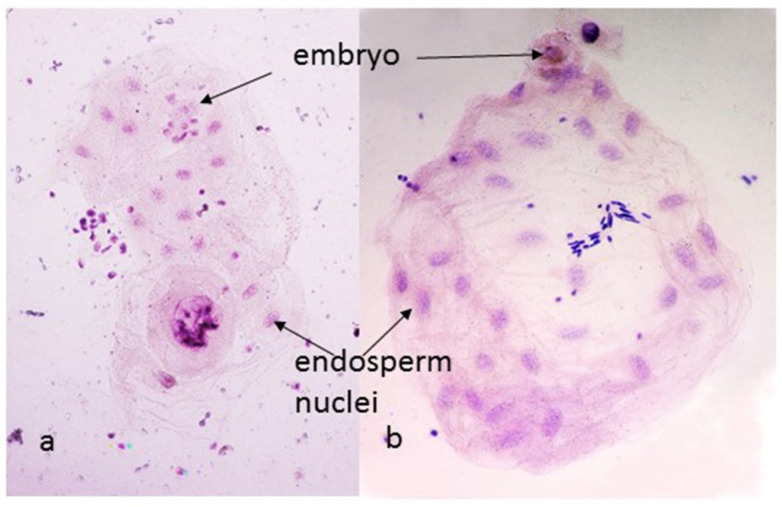
Endosperms from (*kot*)Pavon Dt 1BL (a) and (*aes*)Pavon (b), 24 h after pollination. The endosperm with the *Ae. kotschyi* cytoplasm and 1BS arm missing (a) shows a single cell embryo and 16 endosperm nuclei, indicating four rounds of division; with 1BS arm present (b) the embryo has gone through one additional round of division and the endosperm has 32 nuclei.

Because of the dramatic effect of the combination of the cytoplasm and the 1BS chromosome arm on seed size, the rate of early endosperm divisions was observed at 24, 48 and 72 h after pollination in (*aes*)Pavon, (*kot*)Pavon and (*kot*)Pavon Dt1BL. The effect on the embryo development could not be reliably tested as during dissection of young endosperms embryos are often lost. In the coenocytic stage of endosperm development only nuclei divide, and the rate of divisions is rapid and synchronized, with five to six nuclear divisions producing 32–64 endosperm nuclei in the first 24 h (Figure 3). Later, the division rate slows down, especially at and after cellularization at ca. 72 h post pollination. In total, 125 endosperms were analyzed: 55 at 24 h post-pollination 42 at 48 h post pollination and 28 at 72 h. At 24 h, the endosperms with *Ae. kotschyi* cytoplasm and 1BS missing averaged 14.7 nuclei vs. 30.1 for the 1BS arm present. For 48 and 72 h post pollination the comparable numbers were 125.1 vs. 284.3 and 1733.3 vs. >4000 (numbers of nuclei at 72 h were estimated). Endosperms with the *Ae. kotschyi* cytoplasm and 1BS missing lagged by more than one round of nuclear divisions per 24 h. Perhaps more interesting observation was the presence of two cases, among the 82 embryos examined, where dividing embryos with three and four cells each were clearly identifiable, but there was no endosperm, only a single diffuse nucleus in its place. This suggests that fertilization of the egg (or induction of egg divisions without fertilization) without fertilization of the double nucleus of the embryo sack also takes place in this system.

### 3.3. Genetic Mapping of Factors Controlling Parthenogenesis

**Figure 4:**
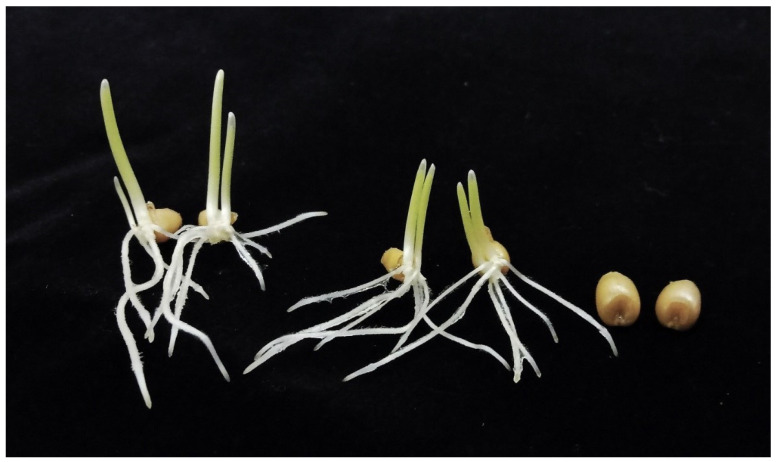
Twin seedlings and embryo-less kernels among the BC_1_ progeny of (*kot*)Joker x (Pavon 1RS.1BL_jok_ x Joker).

To select individuals suitable for genetic mapping (diploid homozygotes 1RS.1BL), 942 backcross progeny from the cross (*kot*)Joker x (Joker x Pavon 1RS.1BL_jok_) were screened cytologically. Of these, 153 were twin seedlings, 351 were haploid, 297 were diploid heterozygotes 20” + 1RS.1BL + 1B, and 141 were diploid homozygotes 20” + 1RS.1BL_jok_”. Additionally, there were 70 embryo-less kernels. With twins included, the haploid frequency was 53.5%. Among twin seedlings, the coleoptiles appeared to be of about equal size in 40; and 113 were clearly unequal (Figure 4). One root each from each part of a twin was collected from a sample of such seedlings; 38 were scored for the ploidy level and 16 were karyotyped for their chromosome constitution. There were 28 haplo-diplo and 10 haplo-haplo twins (assuming that indeed, roots were always collected from each part of a seedling). Among the 16 karyotyped diploids, seven were homozygous 1RS.1BL and nine heterozygous 1RS.1BL + 1B, not a significant deviation from random segregation. Twin seedlings were not included in the mapping population because as it was difficult to reliably separate the diploid and haploid parts.

**Figure 5:**
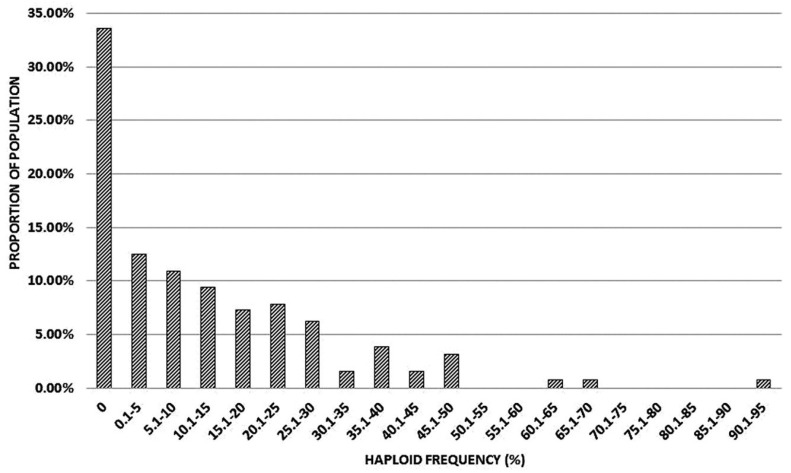
Distribution of haploid frequencies produced by individual members of the mapping population (*kot*)Joker x (Pavon 1RS.1BL_jok_ x Joker).

The rate of parthenogenesis among the members of the mapping population were scored by chromosome counts in seed samples produced by cross-pollination with pollen from various wheats flowering at the time. Haploid frequency of (*kot*)Joker, growing side by side with the mapping population, was 42.5% in a sample of 153 progeny from the maintenance backcross and crosses to various different wheats. This total does not include twin seedlings. Among the mapping population, 2816 progeny were scored with the average progeny sample size of 21.7. Several seed samples were small, either due to incomplete pollination, reduced seed set or a high proportion of non-germinating seed. Overall, the rate of haploid production did not segregate in the 1:1 ratio for no haploids: haploids, as expected for a single parthenogenesis suppressor. Assuming some excess in the “0 haploid” class, presumably due to small sample sizes, and no clear demarcation point between low/intermediate and high haploid frequencies, it appeared likely that at least two loci affecting haploid production segregated in the mapping population (Figure 5).

Among 131 lines of the mapping population genotyped by DArTseq, two lines could not be scored, DNA of one line was of insufficient quality, and genotyping data from one line could not be interpreted, reducing the population to 127. The genotype data for these 127 lines with a total of 8216 markers were obtained. There were 1845 polymorphic markers between Pavon and Joker but 202 markers with more than 20% of missing values in the population were excluded, leaving 1643 markers for multiple rounds of the genetic map construction. After five rounds of ordering and filtering, 1467 markers formed 21 linkage groups corresponding to the 21 chromosomes. Marker coverage for the A, B and C genomes was 563 (38.38%), 718 (48.94%), 186 (12.68%) and the numbers of markers per chromosome ranged from 13 (3D) to 145 (7B). 

**Figure 6:**
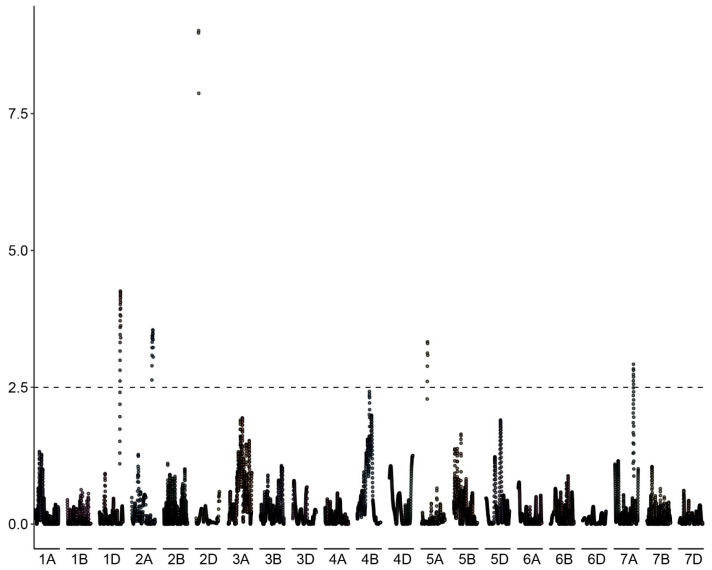
Manhattan plot of the associations between DNA polymorphisms and the haploid production rate in the biparental BC_1_ population (*kot*)Joker x (Pavon 1RS.1BL_jok_ x Joker) with the cytoplasm of *Ae. kotschyi*. A major effect locus appears located on the long arm of chromosome 2D, flanked by markers 541186 and 3020796. Minor effect loci appear likely on chromosomes 1D, 2A, 5A and 7A.

**Figure 7:**
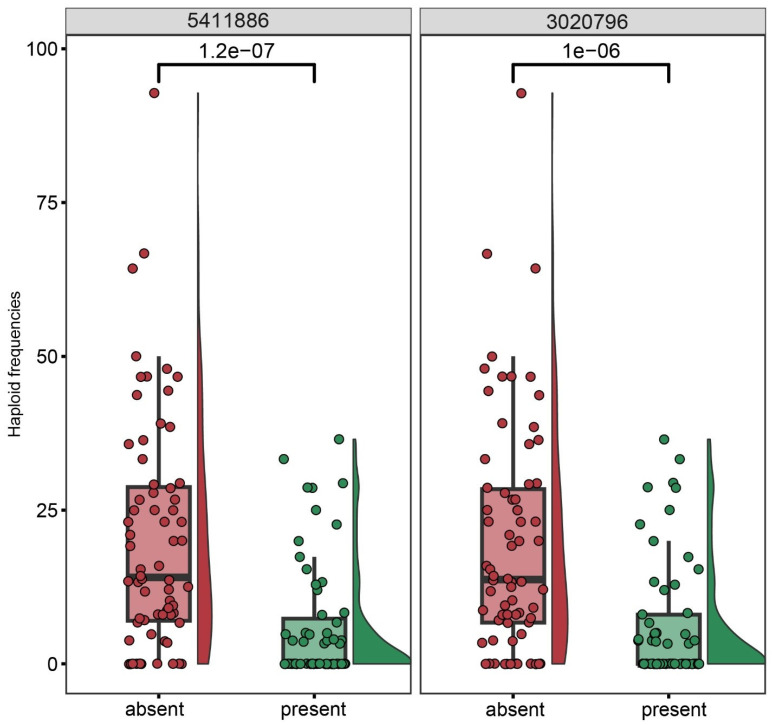
Genetic effects of the two makers flanking the major QTL locus on chromosome 2D from cv. Pavon associated with the rate of haploid production in the biparental BC_1_ population *(kot*)Joker x (Pavon 1RS.1BL_jok_ x Joker) with the cytoplasm of *Ae. kotschyi*.

The proportion of haploids among the progeny of each of the 127 genotyped individuals of the BC_1_ population (*kot*)Joker x (Joker x Pavon 1RS.1BL_jok_) was used as phenotyping data. Five peaks with LOD >2.5 were detected, on chromosomes 2D, 1D, 2A, 5A and 7A (Figure 6). The five loci explained a total of 46.51% of the phenotypic variation in the population (Table 3). The major QTL was located on the long arm of chromosome 2D, with the LOD score 9.01 and contributing 19.34% of the phenotypic variation. The flanking markers of this locus in a region spanning ca. 14 Mbp, from 580 Mbp to 594 Mbp in the Chinese Spring IWGSC reference genome version 2.1 [30]. This locus confers negative effects on the haploid production. Individuals of the mapping population lacking markers flanking this locus showed significantly higher haploid frequencies than lines with the flanking markers present (Figure 7). This locus in Pavon, designated Qhpsu-2D, had a very strong suppressive effect on the haploid production in the tested population. In the genomic region on chromosome 2D delimited by markers 5411886 and 3020796, 205 high-confidence genes are annotated in the IWGSC v2.1 wheat genome (Supplementary Table S1). Future attempts aimed at fine-mapping of this suppressor locus will be crucial to further narrow down the region and reduce the number of potential gene candidates. In addition to the suppressor locus on chromosome 2D, this study identified four other chromosome regions associated with haploid production, all with lower effects than the locus on chromosome 2D. These loci were located on chromosomes 1DS, 2AL, 5AL, and 7AL, with LOD scores of 4.25, 3.54, 3.33, and 2.92, respectively (Table 3). They contributed 8.22%, 7.17%, 6.32%, and 5.44% of the phenotypic variation, respectively. The loci on chromosomes 1D, 2A and 7A were associated with reduced frequency of haploid production; the locus on 5AL conferred a positive effect on haploid production in the population, suggesting its possible role as an enhancer (Supplementary Figure S1). As the size of the genomic intervals for those loci ranges from 0.288 Mbp for the locus on chromosome 1DS to nearly 19 Mbp for the locus on chromosomes 5AL (Supplementary Tables S2–S5), here as well future studies are needed to narrow down the list of potential gene candidates. 3.2.

## 4. Discussion

The *Rf^multi^* locus has been located [7] and identified [31], the candidate genes have been proposed [16,17,18] and there is little doubt that it is the main restorer of male fertility in the cytoplasm of *Ae. kotschyi* in wheat. However, the strange behavior of chromosome 1B_1:6_ raises questions about possible other fertility restoration factors that may be present on the chromosome arm. Numerous DNA sequences encoding *Restorer of Fertility*-like proteins are present in the vicinity of the *Rf^multi^* gene on the short arm of chromosome 1BS in wheat [16,32]. Perhaps some of these are capable of restoring a low level of male fertility. The three engineered chromosomes of 1B, 1B_1:6_, 1B_25:6_, and 1B_35:6_, share the same distal breakpoint from primary recombinant T-6 [9,10]; they differ by the proximal breakpoints. The rye segment removing *Rf^multi^* in 1B_1:6_ is the longest [8]; hence, it removes the largest corresponding wheat segment. The other two engineered chromosomes, 1B_25:6_ and 1B_35:6_, with shorter wheat segments removed, have never shown any anther dehiscence or seed set, while 1B_1:6_ shows it in some seasons, usually during the fall when greenhouse conditions are milder (flowering is usually in late November to mid-December). If indeed some additional *Rf*-like DNA sequences in the vicinity of the *Rf^multi^* locus are responsible for the low level of fertility restoration, all three engineered chromosomes, as well as some recombinants in the T- configuration with breakpoints distal to the main *Rf^multi^* locus, should show the same behavior, but they do not. There are *Rf* -like sequences in the rye 1RS arm [18] but no anther dehiscence or seed set at any level was ever observed in (*kot*)Pavon 1RS.1BL over at least ten generations grown in various seasons. A speculative explanation is that perhaps some *Rf*-like motifs present in 1RS are incapable of restoring even a trace of male fertility on their own, but when combined with some motifs on 1BS, by a fortuitous crossover event, which creates the 1B+1 breakpoint, they generate detectable anther dehiscence and a low seed set when conditions are right.

Observations presented here show clearly that the *Rf^multi^* locus affects more than male fertility and that more than a single gene must be involved. In the absence of a chromosome arm in the 1S position of the B genome (that is, either 1BS or 1RS), the growth rate is seriously reduced, starting as early as the first 24 h of new seed development and continuing through plant maturity. The *Rf^multi^* cannot be directly involved as it is absent from standard 1RS and from engineered 1B chromosomes. All of these chromosomes, even when present in a single dose, produce normally vigorous plants with fully developed grain upon cross-pollination. Either some other loci present on both 1BS and 1RS generate normal vigor or the *Rf^multi^* region, both on 1BS and 1RS but not *Rf^multi^* itself, are involved in interactions with the mitochondrial genome.

The *Rf^multi^* locus has a major effect on the pollen transmission rates of chromosomes 1B and 1RS.1BL, or, rather, on the competitive advantage/disadvantage of pollen grains carrying one of these chromosomes, favoring pollen with the locus present. It is possible that different transmission rates are a consequence of differences in pollen tube growth rates. A restorer to the *Ae. juvenalis* cytoplasm in wheat was implicated in pollen germination and vegetative growth and proposed as *Rfd1* [20]. At the same time, the transmission rates of individual chromosomes (or, rather, their competitive advantage/disadvantage) depend to some extent on the allelic composition of the competing chromosome arms. Pollen bearing the 1RS.1BL translocation can be competitive with pollen carrying 1BS from wheats other than Pavon (see Table 2), producing an almost random transmission rate of the translocation. However, factors other than *Rf^multi^* itself (or, rather, the region in its immediate vicinity as present in engineered chromosomes 1B and 1R), must also be involved. Interestingly, the transmission rates of the 1RS.1BL translocation from heterozygotes with 1B observed here are in direct contrast to those reported before [33], where preferential retention of 1B (or discrimination against 1RS.1BL) was observed in the eggs. Taking these observations together, the system tested here offers interesting research avenues into the nuclear–mitochondrial interaction going far beyond male sterility.

The combination of the *Ae. kotschyi* cytoplasm and a rye 1RS inducer is well known for haploid production [1,4,5] but it does not only induce parthenogenesis. It clearly affects all aspects of double fertilization in wheat: fertilization of the egg cell without fertilization of the double nucleus in the embryo sack; fertilization of the double nucleus of the embryo sack without fertilization of the egg cell producing embryo-less grain; fertilization of the double nucleus in the embryo sack and induction of the development of the egg without fertilization (parthenogenesis); fertilization of both the egg and a synergid, producing diplo–diplo twins; and fertilization of a synergid and induction of the egg development producing haplo–diplo twins. Haplo–diplo–haplo triplets are occasionally produced, but with two sperm nuclei in the pollen and a normally developed seed (triple fertilization can be ruled out unless two pollen grains are involved). However, the system clearly requires much further study as, at present, it is far from clear if allelic variation for parthenogenesis inducers exists or is obscured by the presence of suppressors and perhaps even enhancers. To this day, the authors have not found a single wheat accession with a recessive allele of *Rf^multi^*. Indeed, some observations are strange: as reported by Lukaszewski [9], three accessions of *T. spelta var. duhamelianum* tested for male fertility restoration were almost completely male sterile in the F_1_ generation with (*kot*)Pavon 1RS.1BL. *T. spelta var. duhamelianum* was the only non-restoring wheat reported [3,4,7] but detailed data on that observation are no longer available from the authors. The three accessions tested here, which produced almost male sterile F_1_, restored male fertility in a normal fashion (that is, at the same level as any other 1B chromosome tested) in generations from BC_1_ onward. When single chromosome substitutions of 1Bs from these three accessions into cv. Pavon (BC_7_) were crossed as male to (*kot*)Joker, one F_1_ set 2.22 seeds per spikelet, typical for the restoration level by one copy of *Rf^multi^*. Another F_1_ hybrid set 0.17 seeds per spikelet, and the third one was completely male sterile. Similarly puzzling effects were observed in three wheats from the Wheat 10+ genomes project: cvs. Arinalfor, CDC Stanley, and Mace [34]. Their F_1_ hybrids with (*kot*)Pavon 1RS.1BL were almost completely male sterile (seed set per spikelet >0.1), while their euplasmic control hybrids with (*aes*)Pavon 1RS.1BL were normally fertile (seed set 1.65 to 2.94 per spikelet), suggesting the absence of fertility restoration to the cytoplasm of *Ae. kotschyi.* However, in BC_1_ with the same chromosome constitution, 1B + 1RS.1BL and in the *Ae. kotschyi* cytoplasm, the seed set was around 65% (ca. 2.0 per spikelet), typical of fertility restoration by a single dose of *Rf^multi^*. At present, the authors do not have any sensible explanation for this behavior.

To start untangling the issues associated with the effects of the *Ae. kotschyi* cytoplasm in wheat, an attempt was made to detect and tag suppressors of parthenogenesis. The result appeared more complicated than we had hoped for. It is obvious that haploid frequencies scored here carry a certain error, especially for low frequencies. As much as a small sample appears adequate to determine a high haploid frequency, with haploids absent, there is no upper limit on the minimum sample size. With a 10% assumed haploid frequency, the minimum sample size for a 95% probability of observing haploids is 28.4; this drops to 13.6 for a 20% haploid frequency. Here, the average sample size for the entire population was 21.7. Moreover, some effect of the pollinator on haploid frequencies has been reported [1]. This effect was ignored here as it was not possible to grow a sufficient number of the same pollinator to produce pollen over the entire flowering time of the mapping population members. If there was a pollinator effect here, it likely was minor; no major differences were observed between haploid frequencies in the maintenance backcross of (*kot*)Joker and crosses of (*kot*)Joker with several different wheats made during the same period.

In the biparental mapping population Joker x Pavon 1RS.1BL_jok_, five genomic regions appeared to be significantly associated with haploid production. Four of those, located on the chromosome arms 1DS, 2DL, 2AL, and 7AL, suppressed haploid production. One locus, located on chromosome 5AL, appeared to enhance it. The main effect of suppression was strongly associated with the region on chromosome 2DL. Chromosome arm 1BS with its known parthenogenesis suppressor was absent in the mapping population as all individuals were homozygous for the 1RS.1BL translocation from cv. Joker. At present, it is not clear if genetic variation exists for either the inducer (1RS) or the 1BS suppressor. Some casual observations of the effects of various 1BS arms, including data from the Tsunewaki group [1,2,3,4,7,11,26,33], suggest that chromosome arms 1BS may carry different alleles at the suppressor locus, which may either completely inhibit haploid production, such as 1BS of Pavon, or only partially so, as 1BS of cv. Chinese Spring. Tsunewaki et al. [1] performed a monosomic analysis of the haploid induction rate in a cross (*umbelulata*)Salmon x (CS monosomics x Salmon). Haploid frequencies ranged from 5 to 38%. With only two progenies per combination scored and with small samples, there was no conclusive evidence of a strong suppressor or suppressors. However, it appeared quite convincing that 1BS of Chinese Spring did not completely suppress haploid production. The fact that wheats such as Salmon, Joker, and Aurora [11] can produce haploids at a rate of 90% and higher clearly suggests that allelic variation at the suppressor loci, other than that on 1BS, is present. How frequent is unclear at the moment.

Tsunewaki and Mukai [4] proposed the designations *Spg* (*suppression of parthenogenesis*) and *Ptg* (*parthenogenesis*) for loci involved in haploid production. The former was located on chromosome arm 1BS; the latter on rye chromosome arm 1RS. They also suspected that the wheat line, Salmon, may carry a recessive gene that promotes haploid formation, or that Chinese Spring, used in their studies, carried a suppressor, “or both”. This study suggests that the worst-case scenario suspected by Tsunewaki and Mukai [4] may in fact be true: other *Spg* loci are present in wheat genomes. Five genome regions affecting haploid production were identified here (Table 3). All four suppressor loci were contributed by cv. Pavon; the presumed enhancer locus originated from cv. Joker. Perhaps this is the enhancer locus speculated on by Tsunewaki and Mukai [4] or perhaps some other effect, such as heterozygosity for two alleles at the locus. To follow the convention proposed by Tsunewaki and Mukai [4], the suppression locus on chromosome 1B should be renamed *Spg-1B*, and those detected here would be *Spg-1D*, *Spg-2D*, and *Spg-7A*. If the locus on 5A indeed enhances haploid production, it is proposed to name *it Epg-5A* (*enhancement of parthenogenesis*). Data collected here suggest that a very high frequency of haploids among progeny is a consequence of a combination of an effective inducer, on rye chromosome arm 1RS, an enhancer on chromosome 5A, and the absence of suppressors.

It is not clear if allelic variation of the parthenogenesis inducer, *Ptg*, exists or if this inducer is ubiquitous. Clearly, 1RS arms present in Salmon, Joker, and triticale Presto carry highly effective inducers. Based on data by Mukai [11] the 1RS.1BL translocation in cv. Aurora also carries *Ptg*. If so, then very likely cv. Kavkaz and all Veery lines of CIMMYT, also carry such an inducer, as they all originate from the same rye source [35]. Aurora and Kavkaz carry 1RS.1BL translocations originating from the same source, cv. Neuzucht [21] and appear identical [22]. The fact that wheats Salmon, Aurora, Joker, and triticale Presto are capable of producing over 90% haploid progeny in some seasons suggests that they may also carry the enhancer on 5AS; wheats Salmon, Joker, and Aurora do not carry the suppressor on 2D; cv. Presto does not have it by virtue of its chromosome constitution. Beyond that, it is impossible to speculate on the extent allelic variation of the other loci affecting haploid frequency, but the fact that genetic mapping was possible in this study indicates that such variation does exist. Unclear are the frequencies of various alleles and their individual contributions. This would require a considerable effort of setting up suitable genetic stocks for tests, unless credible haplotypes are established for the loci involved, to select genetic backgrounds free of suppressors. The authors have created a large set of introgressions of 1RS chromosome arms, mostly as 1RS.1BL translocations but also as whole chromosome substitutions and centric translocations to other group-1 chromosomes, but only in cv. Pavon, which, with its array of suppressors, is quite unsuitable for tests. Chinese Spring, with its wide range of cytogenetic stocks would be an obvious choice, but it carries the 2D suppressing haplotype. Cv. Joker is not a sensible option as it requires a very long vernalization period, which makes two growth cycles per year almost impossible.

## 5. Conclusions

Apart from confirming the well-known fact that the cytoplasm of *Ae. kotschyi* induces cytoplasmic male sterility and parthenogenesis in wheat, this study shows that it also engages in numerous additional interactions with the nuclear genome. These interactions affect such characteristics as growth rate, starting from the earliest stages of seed development all the way to seed and plant maturity, and quite likely also extend to pollen tube growth, with a direct impact on chromosome transmission rates. These interactions involve not only the ubiquitous male fertility restorer *Rf^multi^* on chromosome arm 1BS but also other loci on this arm as well as on the rye 1RS arm. Some of these effects appear to be mitigated by the allelic constitution of the chromosome arm 1BS itself; so far, the effects of different 1RS arms could not be tested. The nuclear–cytoplasmic interactions not only induce parthenogenesis but affect all aspects of double fertilization in wheat. Two new inducers of parthenogenesis have been identified here, but the general frequency of such inducers is far from clear. The nuclear genomes of some wheats carry suppressors of parthenogenesis; genetic mapping in one population has shown that several suppressors, and possibly an enhancer, can be present. The strongest suppression effect was associated with a segment of wheat chromosome arm 2DL. As the frequencies and distribution of parthenogenesis suppressors remain unknown, the frequencies and effectiveness of inducers cannot be easily determined.

## Figures and Tables

**Table 1:** Transmission rates of centric wheat-rye translocations from heterozygotes through pollen and eggs, depending on the cytoplasm. *aes*: cytoplasm of *T. aestivum*; *kot*: cytoplasm of *Ae. kotschyi*. Egg transmission was not tested in all combinations as full seed set in cross-pollinations demonstrated the absence of gametic selection.

Chromosome Constitution	Cytoplasms of Cross Combinations	Transmission Tested (♀/♂)	Numbers of Progeny Tested	Transmission Rate of Translocations
1RS.1BL + 1B	*aes x aes*	♂	28	46.4%
	*aes x kot*	♂	99	0.0%
	*kot x kot*	♂	53	0.0%
	*kot x aes*	♂	26	46.1%
	*kot x aes*	♀	27	44.4%
	*kot x kot*	♀	27	51.8%
2BS.2RL + 2B	*aes x aes*	♂	33	51.5%
	*aes x kot*	♂	55	52.7%
	*kot x kot*	♂	30	46.6%
	*kot x aes*	♂	32	46.8%
	*kot x aes*	♀	27	51.8%
	*kot x kot*	♀	27	48.1%
5RS.5AL + 5A	*aes x aes*	♂	30	43.3%
	*aes x kot*	♂	47	40.4%
	*kot x aes*	♂	19	42.1%
	*kot x kot*	♂	55	45.5%

**Table 2:** Male transmission rates of chromosomes 1RS.1BL and 1B with the locus *Rf^multi^* present or absent via pollen with cytoplasms of *T. aestivum* and *Ae. kotschyi* in cv. Pavon 76, and several other assorted wheats. In cv. (*kot*)Pavon the female transmission rates of 1B*Rf* and 1RS*Rf* were 14/29 and 15/29, respectively.

Cytoplasm/Cultivar	*Rf* Location	1B	1RS.1BL	Total
*(aes*)Pavon	1RS*Rf*.1BL + 1B*rf*	9	9	18
(*aes*)Pavon	1RS*rf*.1BL + 1B*Rf*	11	10	21
(*kot*)Pavon	1RS*Rf*.1BL+ 1B*Rf*	38	15	53
(*kot*)Pavon	1RS*Rf*.1BL + 1B*rf*	29	32	61
(*kot*)Pavon	1RS*rf*.1BL + 1B*Rf*	108	7	115
(*kot*)Assorted	1RS*rf*.1BL + 1B*Rf*	50	28	78

**Table 3:** Flanking DNA markers and the effects of QTLs associated with the haploid production rate in the biparental BC_1_ population (*kot*)Joker x (Pavon 1RS.1BL_jok_ x Joker). PVE (%): phenotypic variation explained by QTL. Add: estimated additive effect of QTL. Positive values represent the QTL effects contributed by cv. Pavon. The negative value represents the QTL effect contributed by cv. Joker.

Chromo-some	Left Marker	Right Marker	LOD	PVE (%)	Add	Position of Left Marker	Position of Right Marker	Length of the Interval (Mbp)
2DL	5411886	3020796	9.015	19.340	15.827	594558878	580305266	14.3
1DS	6035557	4539549	4.257	8.222	10.450	20403319	20691938	0.29
2AL	4910904	4910982	3.548	7.180	9.930	63318624	48493438	14.8
5AL	1203412	1141822	3.330	6.325	−9.157	633464714	614653907	18.8
7AL	2261898	1008272	2.920	5.441	8.394	661807951	676342791	14.5

## Data Availability

All data and materials are available from A.J.L., with a standard transfer agreement.

## Supplementary Materials

The following supporting information can be downloaded at: https://www.mdpi.com/article/10.3390/genes15070855/s1, Figure S1. Genetic effects of the makers flanking the QTL loci associated with haploid production in on chromosomes 1D, 2A, 5A and 7A *(kot*)Joker x (Pavon 1RS.1BL_jok_ x Joker) with the cytoplasm of *Ae. kotschyi*. Table S1. Genes present in the genomic interval on the chromosome 2DL. Table S2. Genes present in the genomic interval on the chromosome 1DS. Table S3. Genes present in the genomic interval on the chromosome 2AL. Table S4. Genes present in the genomic interval on the chromosome 5AL. Table S5. Genes present in the genomic interval on the chromosome 7AL.
