# Supplementary material for: New Observations of the Effects of the Cytoplasm of Aegilops kotschyi Boiss. in Bread Wheat Triticum aestivum L
Source: Genes (Basel). 2024 Jun 28;15(7):855. doi: 10.3390/genes15070855 (PMC11275946; doi:10.3390/genes15070855)
Supplement: Supplementary file 1 [file genes-15-00855-s001.zip › Supplementary Figure.pdf]

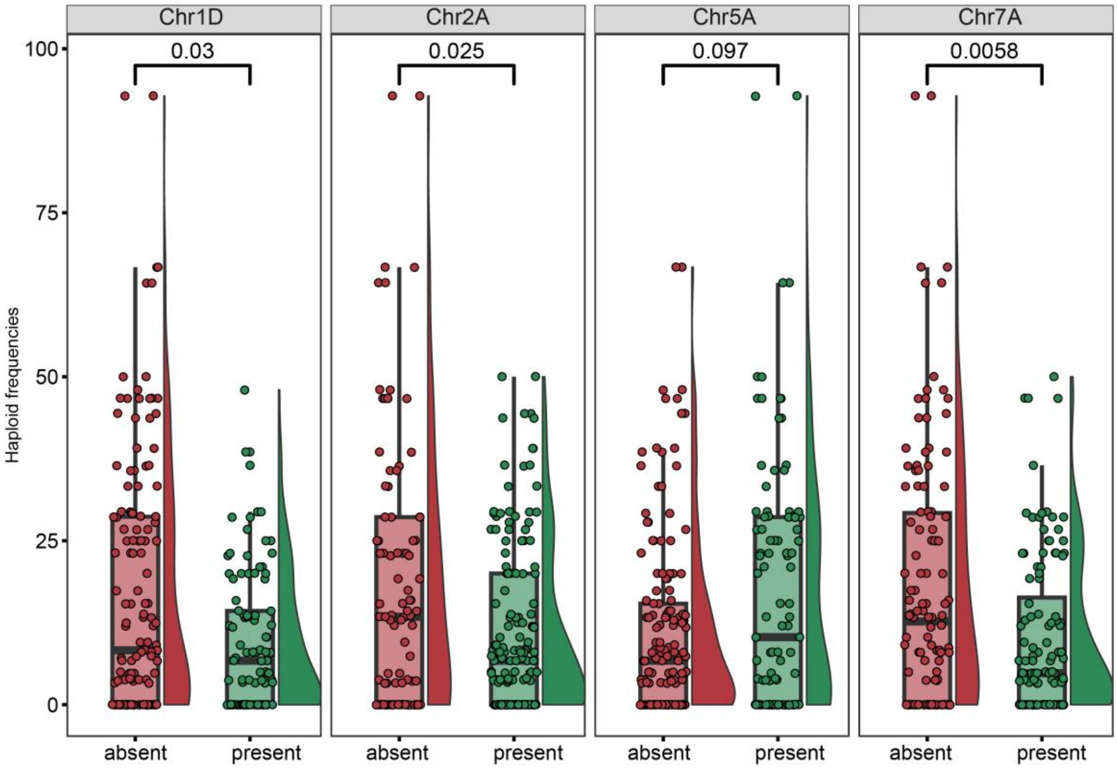

Figure S1. Genetic effects of the makers flanking the QTL loci associated with haploid production in on chromosomes 1D, 2A, 5A and 7A *(kot)*Joker x (Pavon 1RS.1BL$_{jok}$ x Joker) with the cytoplasm of *Ae. kotschyi*.
